# Pulsed Laser and Atomic Layer Deposition of CMOS-Compatible Vanadium Dioxide: Enabling Ultrathin Phase-Change Films

**DOI:** 10.1021/acsaelm.5c01132

**Published:** 2025-07-02

**Authors:** Anna Varini, Cyrille Masserey, Vanessa Conti, Zahra Saadat Somaehsofla, Ehsan Ansari, Igor Stolichnov, Adrian M. Ionescu

**Affiliations:** Nanoelectronic Devices Laboratory, 27218 EPFL, Lausanne 1015, Switzerland

**Keywords:** vanadium dioxide (VO_2_), metal−insulator transition, pulsed laser deposition (PLD), atomic layer deposition (ALD), ultrathin VO_2_ layers, polycrystalline VO_2_ growth dynamics, CMOS-compatible, phase-change, switching devices

## Abstract

Vanadium dioxide (VO_2_), a well-known Mott insulator, is a highly studied electronic material with promising applications in information processing and storage, including neuromorphic and brain-inspired electronics, high-frequency reconfigurable electronics, optoelectronic modulators, sensors, and smart windows with thermal regulation. While epitaxial VO_2_ layers exhibit exceptional properties, such as a sharp and abrupt conductivity change at the metal–insulator transition, fabricating polycrystalline VO_2_ films on silicon substrates often involves trade-offs in transport characteristics and switching performance, especially for ultrathin layers required in advanced gate applications. In this study, we explore the growth dynamics of VO_2_ films on standard CMOS-compatible wet-oxidized silicon wafers by using two established deposition techniques: pulsed laser deposition (PLD) and atomic layer deposition (ALD). VO_2_ films, ranging in thickness from 200 nm to less than 10 nm, were systematically characterized through structural and electrical analyses to optimize key growth parameters. In this study, the temperature and pressure were identified as the key factors influencing the morphology and quality of switching in VO2 films. The growth dynamics and optimal growth conditions across the entire thickness range are discussed in detail. PLD and ALD offer distinct advantages: PLD enables the formation of high-density films, while ALD allows for conformal deposition on complex 3D structures. We demonstrate that both methods can successfully produce ultrathin VO_2_ layers down to 6–8 nm with functional properties suitable for practical applications, provided that growth parameters are carefully optimized. This work underscores the potential of VO_2_ for fully CMOS-compatible phase-change switching devices and provides valuable insights into optimizing growth processes for polycrystalline VO_2_ films grown with different techniques, including widely used magnetron sputtering.

## Graphical abstract

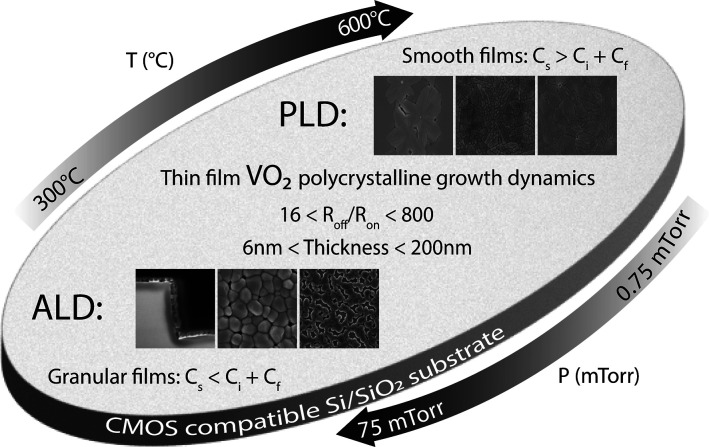

## Introduction

Vanadium dioxide (VO_2_) is a fascinating material in electronics due to its metal–insulator transition (MIT) near room temperature (∼68 °C), where it switches from a low-temperature insulating phase to a high-temperature metallic phase. This transition is sharp and reversible and can be triggered thermally, electrically, optically, or even by strain. VO_2_ thin films demonstrate a reversible change in electrical, optical, and thermal properties^1–5^ that makes them interesting candidates for integration in the wide range of optical and electronic devices, such as switches, modulators, reconfigurable radio frequency devices, memories, transistors,^6–10^ and in different types of sensors.^11–14^ For example, by application of the appropriate strain, the lattice structure of the VO_2_ material can be changed, resulting in a phase transition. Diverse stimuli including temperature, strain, optical, and electric field control can drive the semiconductor-to-metal phase transformation characteristics of VO_2_. The reversible change of VO_2_ properties is based on the phase transition mechanism that is currently explained by combination of Mott-Hubbard phase transition driven by strong electron correlation and the Peierls phase transition driven by the change of the lattice structure.^15–18^ Experimental understanding of the stimulus response of the VO_2_ film to light fields, electrostatic fields, terahertz pulses, or stresses may also complement the understanding of MIT mechanisms.^19^ While there are multiple methods of growing VO_2_ (Sol–gel, Hydrothermal, MBE, MOCVD, chemical vapor deposition (CVD), Sputtering, Magnetron, pulsed laser deposition (PLD), and atomic layer deposition (ALD)), it is difficult to maintain high-purity and high-quality VO_2_ films. The crystal structure and purity of VO_2_ will vary depending on the preparation method, including VO_2_ (R), VO_2_ (M1), VO_2_ (M2), VO_2_ (A), VO_2_ (B), VO_2_ (C), VO_2_ (D), VO_2_ (T), etc.^20,21^ Moreover, for the integration in the CMOS domain, the VO_2_ thin film has to be grown on standard Si wafers with a SiO_2_ top layer that has intrinsic built-in strain and thermal expansion coefficient and lattice mismatch with VO_2_. According to the current state of the art,^22–28^ thin VO_2_ films bellow 10 nm were so far grown only on TiO_2_ wafers with PLD^29^ and MBE^30^ methods. In the case of thin films grown on Si/SiO_2_ substrates, the crystallization process is much more complex, and the presence of grain boundaries and roughness associated with the grain nonuniformity makes the growth of ultrathin films very challenging. While VO_2_ films grown on Si/SiO_2_ substrates are polycrystalline and demonstrate a smaller switching ratio compared to the epitaxial films grown on lattice matching substrates such as sapphire, TiO_2_, or Mica,^29,31,32^ their performance is sufficient for a number of applications, including sensors and other devices.^33–35^ To optimize the material properties for practical applications, it is essential to understand the growth mechanisms of polycrystalline VO_2_ films and how the resulting morphology influences the device performance at a given thickness. A detailed understanding of these mechanisms, coupled with precise control over growth parameters, will enable a more effective design of future applications based on predictable and tunable film properties. It is already known that VO_2_ properties are strongly affected by the substrate temperature, reaction gas composition, gas flow rate, and other factors that have to be well controlled. However, due to a wide range of possible VO_2_ applications and a wide range of growth techniques, reports are often focused on device performance, and film thicknesses are not always properly documented. The present work aims at addressing this issue by reporting specific mechanisms of thin film formation for different thicknesses and on how thickness itself influences film properties. In this work, we use a Si/SiO_2_ CMOS-compatible substrate and demonstrate how control of growth parameters in PLD and ALD recipes influences thin film growth dynamics and resulting morphology as well as switching properties for the range of thickness relevant for industrial integration. Interestingly, we have observed that IMT and MIT values are shifting toward higher values in the films below 100 nm. We attribute this shift to the intrinsic strain of the SiO_2_ layer and the thermal expansion coefficient mismatch of SiO_2_ and VO_2_. This result alone demonstrates doping-like tuning of switching temperature. Gang Xu et al.^36^ used radiofrequency reactive magnetron sputtering for epitaxial growth of VO_2_ thin films on an α-Al_2_O_3_ (0001) sapphire substrate and investigated the 3–150 nm film thickness effect on optical properties. Their results showed that as the film thickness decreased, the crystal metal phase transition temperature of the VO_2_ thin films significantly decreased. In our case of PLD and ALD non-epitaxial VO_2_ growth, the phase transition temperature shifts to the opposite direction and with both IMT and MIT increasing for thinner films. This difference can be explained by the fact that the α-Al_2_O_3_ (0001) substrate has a matching lattice structure and distortion will increase for thicker films, while in our case, initial distortion will have less of an effect for thicker films. Jiang et al.^37^ also found that by accurately adjusting the oxygen flow ratio without doping elements, the phase transition temperature can be controlled between 46 and 72 °C for VO_2_ thin films synthesized on quartz glass. Similarly, our work contributes to our understanding of the phase transition temperature shift for the wide range of VO_2_ thin films grown on CMOS-compatible wafers. Finally, one of the most significant achievements of our work is the successful fabrication of continuous VO_2_ films with thicknesses below 10 nm using both PLD and ALD methods^38–41^ —a result that, to the best of our knowledge, has not been previously demonstrated. There is lack of detailed research on the film formation mechanism for PLD and ALD methods; however, the challenge of growing continuous films below 10 nm of thickness is well documented and normally attributed to the initial growth of island-like particles (formation and growth of crystal nuclei) with subsequent formation of a continuous film at a higher thickness.^42^ We overcame this limitation by employing slow deposition rates and allowing extended time for clusters to coalesce into a continuous film under carefully controlled annealing conditions.

## Design of Experiments and Results

Standard {100} oriented 525 μm Si wafers with 200 nm WetOx (SiO_2_) were used as substrates for all grown films. We report an optimization strategy for both PLD and ALD growth methods that resulted in ultrathin polycrystalline films with switching ratios that are low but useable in VO_2_ devices. Our approach was based on review of previously available data^22–28,38,43^ and narrowing down the variability range of key growth parameters that would definitely result in a continuous switchable film. Rather than minimizing the number of experiments with methods like the Taguchi design, we have manually and carefully varied growth parameters and film thickness to have a better understanding of growth mechanics. This approach led to the observations necessary for understanding and overcoming limitations of island-like discontinuous film formation. Each key parameter was varied in that narrow range, while all other parameters were kept fixed. Such a method might be long and expensive, but it is also the best way to reveal nonlinear dependences of the multiparameter process and the real influence of each evaluated parameter. Below you can find a step by step report on PLD and ALD experiments with selected films that met the initial target of being continuous, pure phase VO2, and switchable with a reasonable On/Off ratio. The quality of resulting films was evaluated by means of four-point probe measurements inside temperature chamber, where temperature was cycled between 40 and 90 °C to determine switching on/off ratio and hysteresis of the film. X-ray diffraction (XRD) measurements were also used to confirm the pure VO_2_ phase or the presence of different V_*x*_O_*y*_ phases. Once the best film was identified for a given variable parameter, that parameter was fixed and investigation was moved into the next influential parameter. The order of testing was decided based on previous results that indicated temperature as the main factor of influence, followed by pressure and laser energy/frequency or fluence of deposition. Growth mechanics observed for different recipes are also discussed in detail.

## PLD Method

PLD is recognized as a fast, clean, and versatile physical vapor deposition technique capable of producing high-crystal-quality films. Its ability to precisely control key parameters—such as laser energy, pulse frequency, substrate temperature, deposition time, total chamber pressure, and partial gas pressure—makes it particularly suitable for growing complex oxides like vanadium dioxide (VO_2_). The fine-tuning of these parameters allows for the careful modulation of film properties, including thickness uniformity, crystallinity, and stoichiometry, all of which are crucial for the growth of continuous thin and ultrathin VO_2_ films and optimizing their metal–insulator transition (MIT) characteristics.

PLD’s unique advantage lies in its ability to maintain stoichiometric transfer from the target to the substrate, even for multicomponent materials, due to the highly energetic nature of the laser-induced ablation plume. This feature ensures the faithful reproduction of the target’s composition in the deposited film, a critical factor for VO_2_, which is sensitive to deviations in vanadium and oxygen ratios. Furthermore, PLD is particularly effective in producing epitaxial and polycrystalline films with minimal contamination, making it an environmentally friendly option compared to CVD or sputtering techniques, which may involve complex precursors or produce harmful byproducts.

Recent advances in PLD have demonstrated its capability to integrate functional dopants into VO_2_, enabling the engineering of its phase-transition temperature and electronic properties. For instance, doping^44–48^ with elements such as chromium (Cr), tungsten (W), niobium (Nb), and germanium (Ge) has been shown to modulate the MIT temperature, expanding the potential for VO_2_ in temperature-tunable devices. This flexibility opens avenues for tailoring VO_2_ for specific applications such as tunable infrared optics, sensors, and neuromorphic computing elements. The straightforward incorporation of dopants via PLD, facilitated by the ability to alternate targets or introduce reactive gases during deposition, provides a promising route for fine-tuning VO_2_’s electrical and optical properties. This capability, combined with the high control over deposition conditions, positions PLD as a state-of-the-art technique for advancing VO_2_-based technologies.

## Temperature Effect

The first most important parameter that influenced PLD growth of VO_2_ was temperature during deposition and additional annealing. In our work, continuous polycrystalline layers with a high on/off ratio started to form at 400 °C. Increasing temperature of deposition has resulted in a different morphology with a more granular and rough film structure with a lower on/off ratio. Figure 1 shows scanning electron microscopy (SEM) micrographs of samples (a–d) grown at a fixed chamber pressure of 7.5 mTorr with 15 sccm of Oxygen, laser frequency of 28 Hz, laser energy 230 mJ, and varied temperatures of 300 °C, 400 °C, 500°C, and 600 °C respectively. Every grown film was characterized by a four-point probe measurement inside the temperature chamber where the temperature was cycled multiple times between 40 and 90 °C. Figure 2 shows a comparison of switching behavior for films grown under identical conditions except for the chamber temperature and XRD results for three switching polycrystalline films. From the comparison of XRD and switching behavior, it can be seen that the sample (b) grown at 400 °C has a VO_2_ phase as well as the highest on/off ratio and lowest hysteresis.

**Figure 1:**
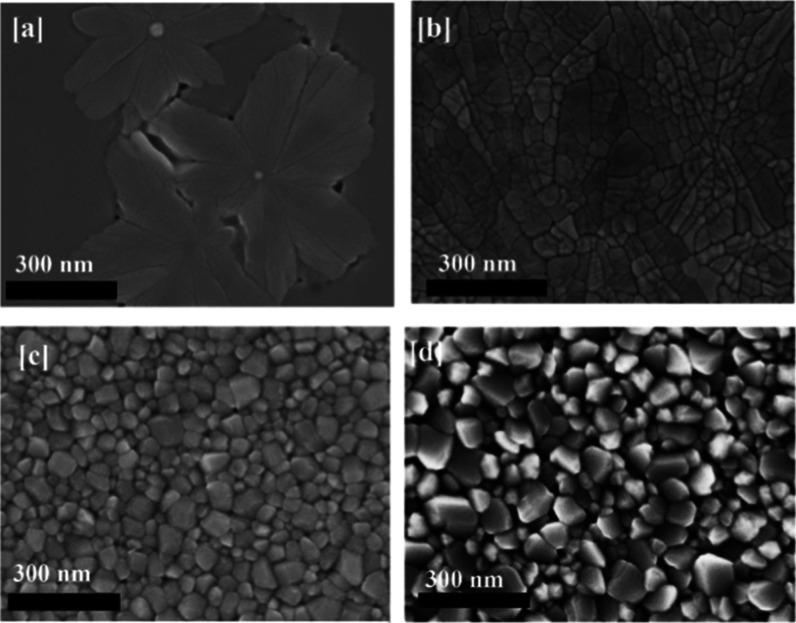
SEM micrographs of VO_2_ films grown at (a) 300 °C, (b) 400 °C, (c) 500 °C, (d) 600 °C. Other growth parameters were fixed for all 4 samples: *P* = 7.5 mTorr, 15 sccm of Oxygen, laser frequency = 28 Hz, laser energy 230 mJ, laser fluence = 1.01 mJ/cm2. Scale bar 300 nm.

**Figure 2:**
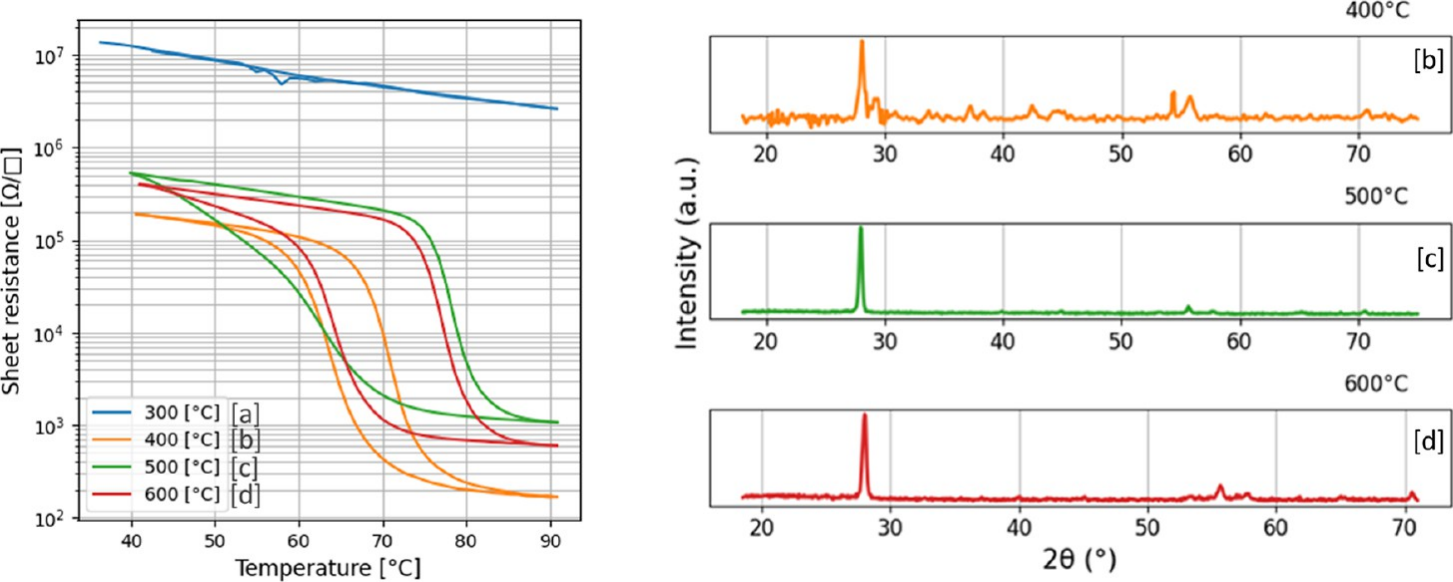
Left: Temperature sweep on samples (a–d) shown in Figure 1 measured with a four-point probe configuration. Right: XRD spectrum for samples (b–d), sample (a) is not measured by XRD due to the amorphous nature of the film.

Therefore, 400 °C was fixed as the optimal temperature, and to further investigate the influence of temperature on film morphology and switching characteristics; sample (b) was annealed for 10 min at 500 and 600 °C. SEM micrographs and respective before and after annealing switching curves for sample (b) from Figure 1 can be seen in Figure 3.

**Figure 3:**
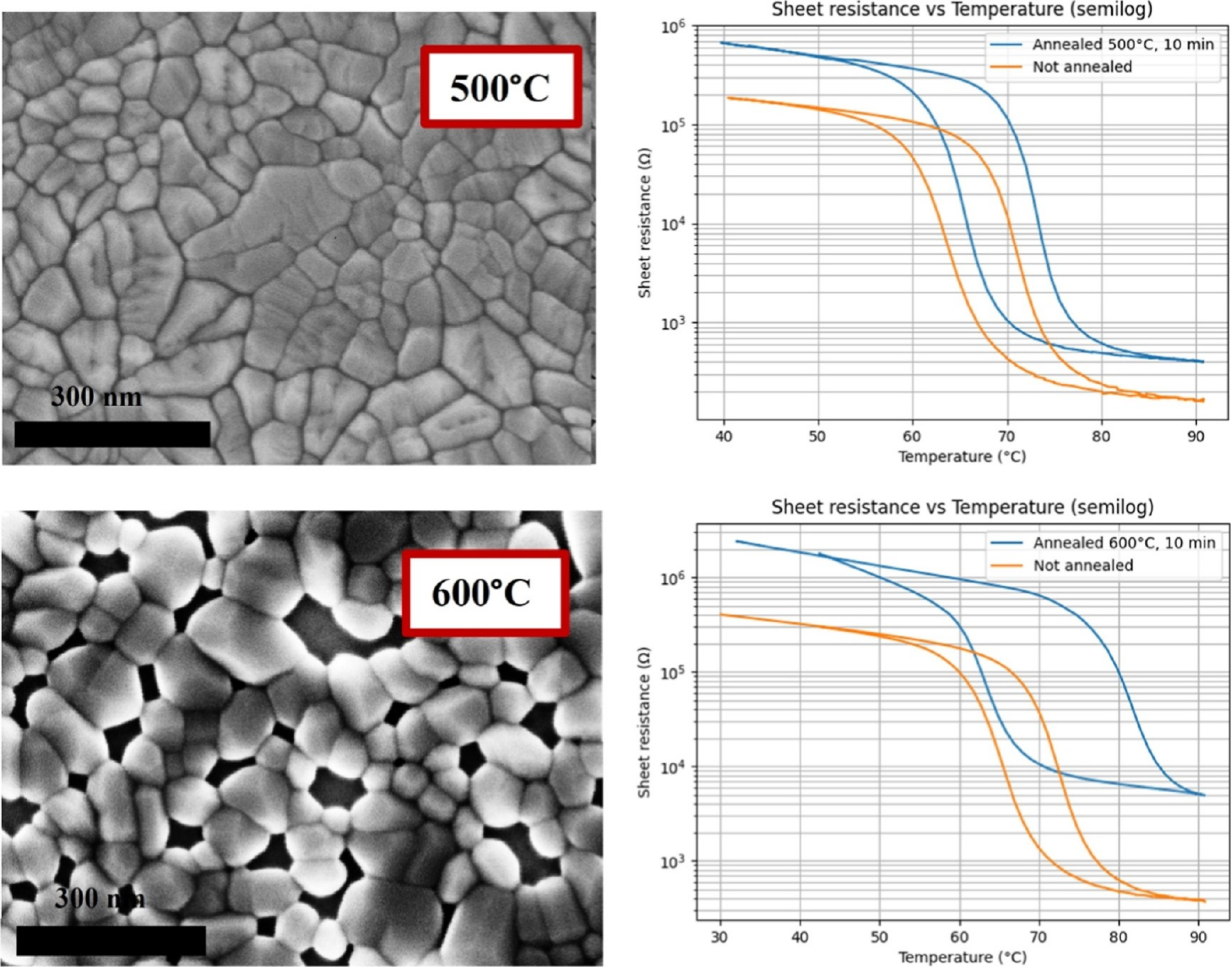
SEM micrographs and respective before and after annealing switching curves for sample (b) from Figure 1. Top part of figure is for annealing at 500 °C for 10 min and bottom part of figure is for annealing at 600 °C for 10 min. Other growth parameters: *P* = 7.5 mTorr, 15 sccm of Oxygen, laser frequency = 28 Hz, laser energy 230 mJ, laser fluence = 1.01 mJ/cm^2^. Scale bar 300 nm.

It can be seen from Figure 3 that annealing at 500 °C has resulted in a higher on/off ratio and smaller hysteresis of the film, while annealing at 600 °C had a negative effect on the film switching, resulting in bigger hysteresis and a smaller on/off ratio. Other annealing temperatures and different film thicknesses were tested. However, we report only the most relevant films for morphology evolution and the highest on/off ratio film that was achieved for 10 min of annealing at 500 °C. The morphology of films annealed at higher temperatures resembles that of the films directly deposited at higher temperatures, but films grown directly at 500 °C have a lower on/off ratio compared to films annealed at 500 °C. All the above observations can be explained by the law of surface energy balance. All the above observations can be explained by the law of surface energy balance: eq 1a describes a smooth film governed by dewetting forces, while eq 1b applies to a film that is stable against dewetting and adopts a rougher, granular morphology. In this latter case, the principle of surface energy minimization drives small grains to coalesce into larger ones, or even form isolated standing grains, as long as the total energy continues to increase.

1a Cs<Ci+Cf

1b Cs>Ci+Cf

where Cs, Ci, and Cf are the substrate, interface, and film surface energies, respectively.^28^

Zhang et al.’s study on qualitative aspects of atomistic processes in the early stage of thin-film growth is directly applicable to our work and provides deep physical insights into kinetic aspects of growth. It is based on classical nucleation theory^49^ as well as on direct visualization of the terrace-step-kink (TSK) model with a scanning tunneling microscope. The diffusion of an adatom on a flat surface or terrace is by far the most important kinetic process in film growth, and this diffusion will strongly depend on initial kinetic energy of the particles ablated with the laser during PLD growth. Figure 1a demonstrates that the total kinetic energy of the process at 300 °C is only enough to initiate randomly occurring nucleation points in the amorphous material. It is well-known that at lower temperatures, surface energy is higher, and it will decrease with increase in temperature. Expectedly in our work, all films grown at temperatures below 450 °C form a smooth flower-like polycrystalline pattern starting from a single nucleation point at the center. Figure 1b is an example of such a smooth morphology for a fully crystallized film grown at 400 °C. The total energy budget that is necessary for full transformation from an amorphous (Figure 1a) to a polycrystalline (Figure 1b) film strongly depends on the amount of material available for film formation, and therefore, continuous fully crystallized ultrathin films can only be formed at a careful kinetic energy balance at rather low temperatures applied for an extended time to allow even and smooth spreading of the deposited material on the wafer surface. As the temperature increases, the smooth flower-like morphology starts to change and better-defined grains start to appear oriented with the longer dimension parallel to the initial grooves. Figure 3 shows two further steps of annealing for the sample in Figure 1b, and it can be seen how increasing the process energy changes long grains into multiple smaller and better-defined grains that still resemble the flower-like original pattern at 500 °C, while complete transformation into a set of randomly oriented grains is visible at 600 °C. Similar observations were reported before by Marvel et al. However, our work expands an understanding of VO_2_ growth dynamics for the wide range of growth conditions and sample thicknesses. Our experimental results agree with the theory of thin film growth mechanics and are consistent for both PLD and ALD samples, which will be further discussed in the ALD part of this paper.

## Pressure Effect

It has been reported that oxygen partial pressure has an impact on the valence state of the final vanadium oxide film and on transition behavior;^50^ however, the magnitude of change in the on/off ratio of switching devices was very small. Sayid Bukhari et al. (2020) suggested that oxygen diffusion may play a crucial role due to the flow rate and reported at what flow rate they started to observe switching behavior and how it was changing. In our work, we have investigated the influence of different O_2_ flow rates in the narrower range that is supposed to yield the best switching behavior; however, we did not observe significant improvement or degradation of film properties. Figure 4 demonstrates variation of oxygen flow rate between 5 and 25 sccm; all other growth parameters were equivalent for these three curves.

**Figure 4:**
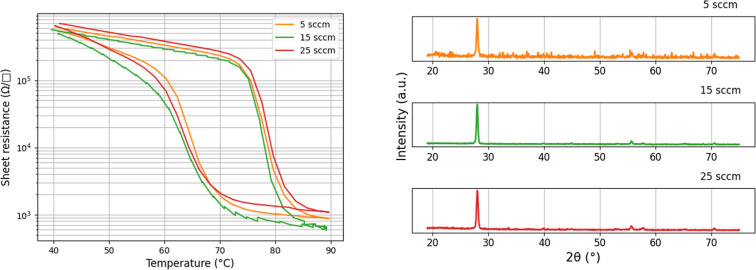
Left: Temperature sweep measured with a four-point probe configuration on samples grown at 5, 15, and 25 sccm of oxygen. Right: respective XRD spectrum for samples grown at 5, 15, and 25 sccm of oxygen. Other growth parameters: *T* = 500 C, *P* = 7.5 mTorr, laser frequency = 28 Hz, laser energy 230 mJ, laser fluence = 1.01 mJ/cm2.

In contrast, a significant influence of the total chamber pressure on the quality of VO_2_ films was observed. Oxygen pressure during both growth and annealing played a critical role in film formation, with a narrow optimal pressure range between 5 and 10 mTorr for PLD growth. Additionally, the total pressure affected the purity of the VO_2_ phase, as indicated by the XRD spectra. A pressure of 6.6 mTorr resulted in the purest VO_2_ films, correlating with the highest switching ratio compared to other samples, as shown in Figure 5.

**Figure 5:**
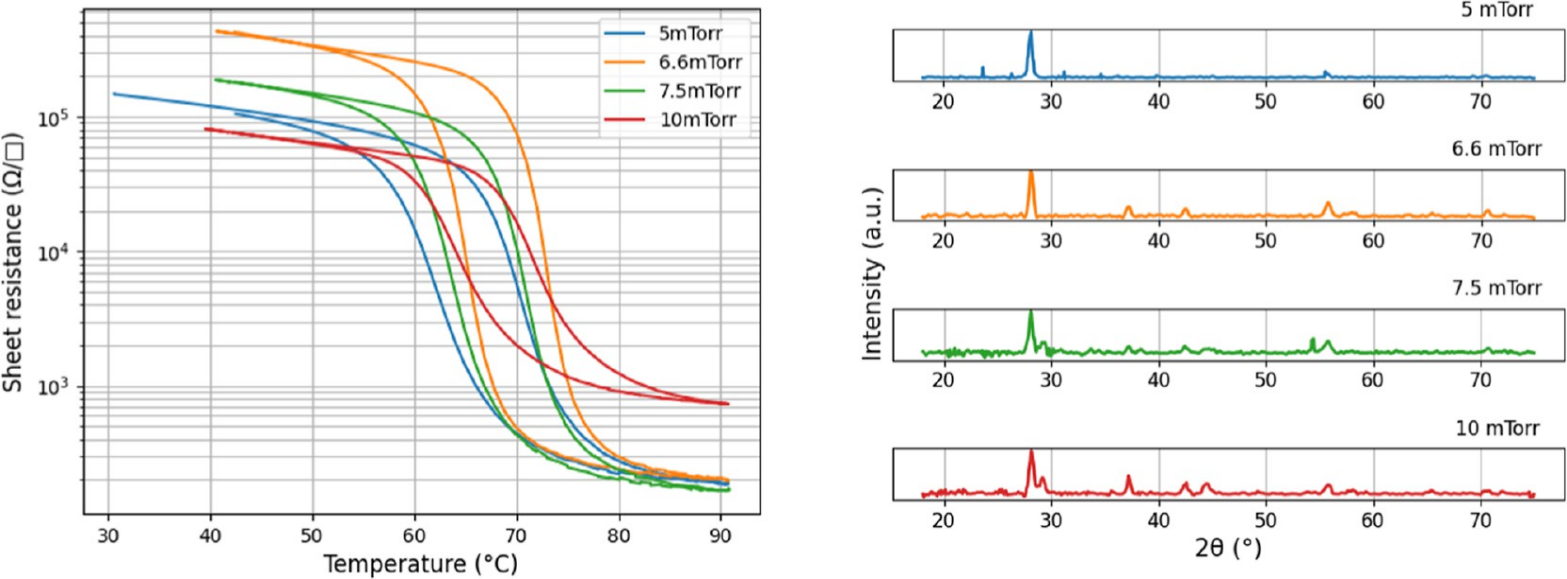
Left: Temperature sweep measured with a four-point probe configuration on samples grown at 5, 6.6, 7.5, and 10 mTorr total chamber pressure. Right: respective XRD spectrum for samples grown at 5, 6.6, 7.5, and 10 mTorr. Other growth parameters: *T* = 400 C, laser frequency = 28 Hz, laser energy 230 mJ, laser fluence = 1.01 mJ/cm2.

## Laser Energy and Frequency

Laser energy and frequency were varied to evaluate their effect on the switching properties of the films. Table 1 has a list of samples evaluated in terms of frequency difference while the energy was kept constant (S1, S2, S3) and samples evaluated in terms of energy while the frequency was kept constant (S2, S4, S5, S6). Figure 6 has thermal switching curves for both sets of samples.

**Table 1:** List of Samples Investigated for the Influence of Frequency and Energy with Complete Description of Used Growth Parameters and Resulting Thickness, On/Off Ratio, Insulating to Metal Transition Temperatures as Well as Results of Raman Spectroscopy with Reported V–O and V–V Bond Peaks Corresponding to Mostly M1 in S1, S2, S3 and Coexistence of the M1/M2 Phase in the V–O Bond in S4, S5, S6

sample	*T* [°C]	*f* [Hz]	O_2_[sccm]	time [s]	*E* [mJ]	*E* [mJ/cm]	*P*[mTorr]	*Z* [nm]	on/off	IMT [°C]	peak V–O	peak V–V
#S1	400	50	15	240	230	1.01	7.5	81	504	70.8	619.3	195
#S2	400	28	15	240	230	1.01	7.5	69	351	70.8	621.8	195.7
#S3	400	10	15	240	230	0.99	7.5	34	15.8	78.5	622.3	195.8
#S4	400	28	15	240	400	1.77	7.5	43	401	73.2	648.5	195.7
#S5	400	28	15	240	300	1.31	7.5	30	472	74.1	654.7	194.97
#S6	400	28	15	240	500	2.23	7.5	40	477	72.4	660.4	194.99

**Figure 6:**
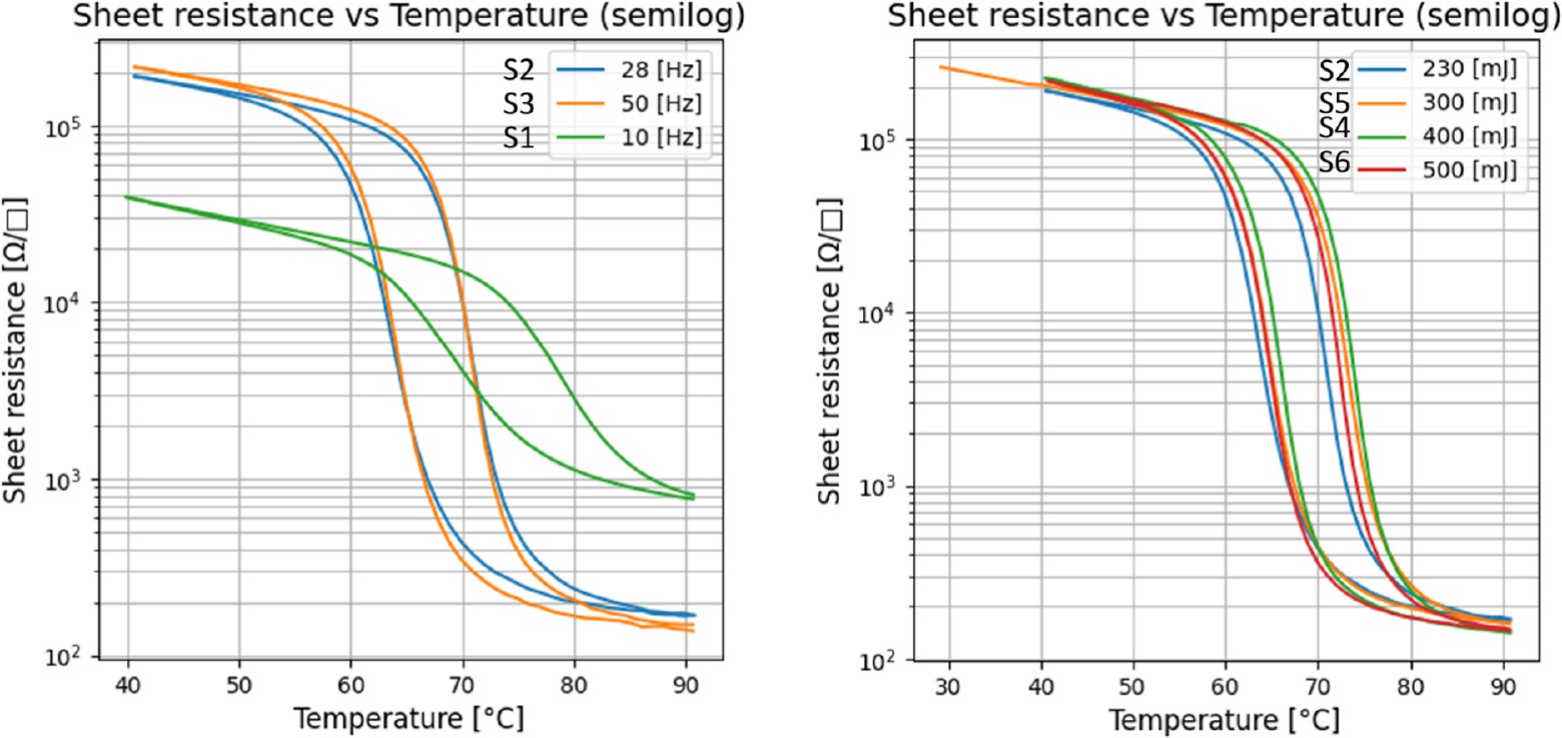
Left: Temperature sweep measured with a four-point probe configuration on samples grown at 10, 28, and 50 Hz. Right: Temperature sweep measured with a four-point probe configuration on samples grown at 230, 300, 400, and 500 mJ. For other growth parameters, see Table 1.

Even though there are no major differences in switching behavior for samples grown at different energies, we undertook further investigation with Raman spectroscopy to reveal the phase balance for samples produced at different energies. Raman spectroscopy was used to compare films grown at 10, 28, and 50 Hz as well as films grown at 230, 300, 400, and 500 [mJ] of laser power. Our results for samples with different frequencies of 10 Hz, 50zH, and 28 Hz show the M1 monoclinic insulating phase with no presence of the M2 phase. However, the M2 phase appeared in the V–O bond with increased energy of the laser. Moreover, the coexistence of the M1-M2 phase of VO2 seems to result in a higher insulating to metal transition temperature (IMT) at around 73 °C.

## Influence of Film Thickness

Based on the experimental results, it can be concluded that the optimal conditions for high-quality VO_2_ thin film growth using the given PLD configuration are 230 mJ laser energy, 28 Hz frequency, 10 sccm oxygen flow, a total chamber pressure of 6.6 mTorr, and a substrate temperature of 400 °C. Additional improvement in the On/Off ratio was observed after a 10 min annealing process at 500 °C. This set of parameters will be referred to as the optimized recipe. Subsequent efforts focused on the growth of ultrathin films and the analysis of the dependence of the On/Off ratio and the insulator–metal transition (IMT) on film thickness.

It has been demonstrated above that the grain structure of the film strongly influences the phase transition in VO_2_. Previous studies have also shown that the insulator–metal transition (IMT) temperature depends on grain growth and deposition temperature.^22^ To minimize these effects, we fixed the growth temperature and maintained a stable surface morphology across varying film thicknesses. This approach allowed us to isolate and assess the specific impact of the film thickness on the switching behavior.

It must be also pointed out that for the nanometer-range variation in film thickness and a few degrees of changes in IMT, the validation of observed dependencies requires statistically representative sets of data. Therefore, we have repeatedly grown multiple wafers under exact same optimized recipe and calculated that our statistics for the On/Off ratio for the optimized recipe are med = 473, mean = 470, std = 76.1 and for optimized plus 10 min annealed at 500 °C are med = 802, mean = 835, std = 160. We have made the same calculations for IMT of the optimized recipe and optimized plus 10 min annealed at 500 °C with med = 72 °C, mean = 71.8 °C, std = 0.449 °C and med = 71.8 °C, mean = 71.8 °C, std = 0.388 °C.

These calculations performed for multiple wafers grown with the optimized recipe have proven reproducible and shown stable growth, and therefore, we have investigated the possibility of scaling down this film thickness by gradually decreasing the time of deposition down to 30 s, which is the minimum allowed time by our PLD machine. Figure 7 demonstrates the observed dependency of the On/Off ratio and IMT temperature for a series of samples grown for different durations and therefore thicknesses according to the optimized recipe. Observed changes can be directly attributed to the presence of unrelaxed thin-film strain originating from lattice mismatch, since thermal and microstructural components were kept constant.

**Figure 7:**
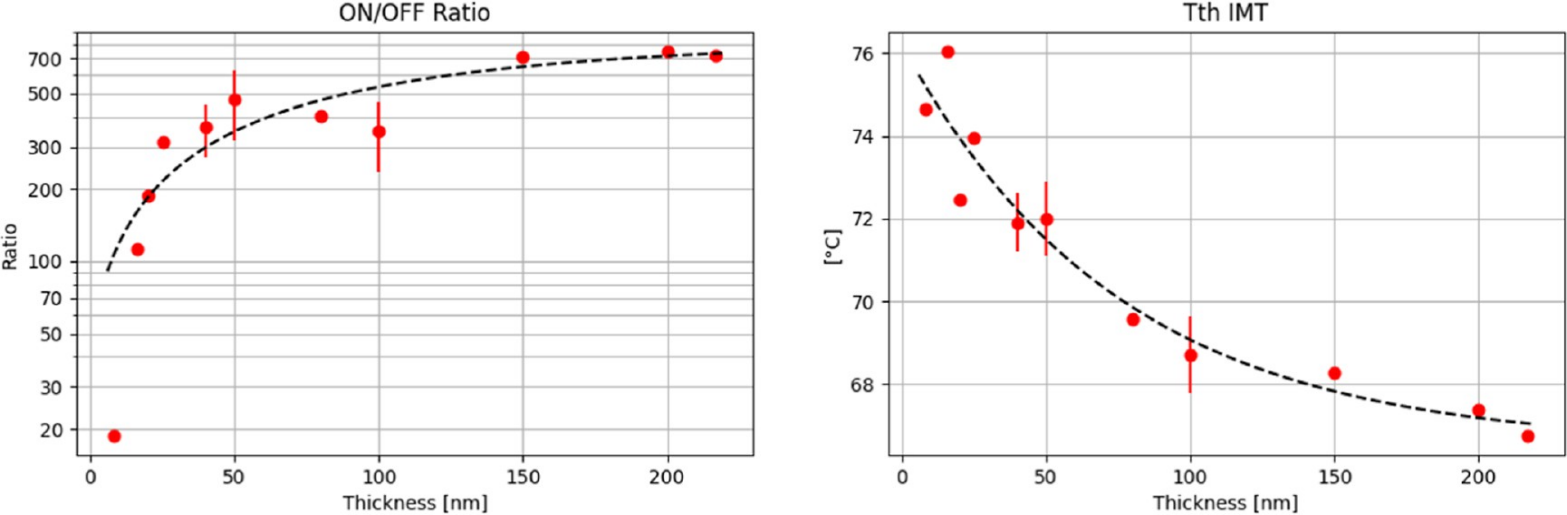
Thermal sweep measurements of VO_2_ films with varying thicknesses grown under optimized PLD conditions (230 mJ laser energy, 28 Hz repetition rate, 10 sccm oxygen flow, 6.6 mTorr chamber pressure, 400 °C substrate temperature). Left: ON/OFF resistance ratio as a function of film thickness, showing an exponential dependence. Right: Threshold temperature for the insulator-to-metal transition (IMT) as a function of film thickness, also following an exponential trend.

## ALD Method

ALD is widely regarded as one of the most precise methods for controlling the thickness of films, offering subnanometer accuracy.^51,52^ Its unique, self-limiting surface reaction mechanism enables layer-by-layer growth, ensuring exceptional uniformity across large areas, even on complex or nanostructured surfaces. This capability is particularly valuable for applications requiring highly conformal coatings, such as in advanced microelectronics and 3D architectures, where uniform coverage on high-aspect-ratio structures is critical. In the context of VO_2_ thin film fabrication, ALD provides several distinct advantages. It allows for the deposition of ultrathin, continuous films with excellent uniformity at the wafer scale, which is essential for achieving consistent electrical and thermal properties across devices. However, since all ALD films are amorphous after deposition, careful annealing conditions are paramount for correct crystallization of the ultrathin VO_2_ film. This work is the first one to report such a process and demonstrate continuous switching below 10 nm.^40,41^ Additionally, the process’s compatibility with CMOS technology makes it highly attractive for integrating VO_2_ into silicon-based platforms, facilitating the development of innovative devices, such as phase-change memory, tunable optical components, and neuromorphic circuits.

For this study, all experiments were conducted using a BENEQ TFS200 ALD system, which offers precise control over the growth parameters, ensuring reproducibility and scalability. The ability to finely tune parameters such as precursor pulse time, purge cycles, and substrate temperature allows for optimization of film properties, including thickness and density that will influence further crystallinity and surface roughness. ALD’s capability to produce conformal, ultrathin layers with high uniformity and its inherent CMOS compatibility underscore its potential as a key enabling technology for next-generation electronic and photonic devices.

ALD depositions reported in this work were run on a Tetrakis ethylmethyl amino vanadium (TEMAV) precursor^38^ that was heated up to 70 °C at the bubbler level, water was used as oxygen source, chamber temperature during deposition was kept at 150 °C apart from one sample grown at 200 °C. Chamber pressure control is not available on our ALD machine and is always set at 4mbar. The thickness of the film was varied by the number of ALD pulses, while pulses were always cycled with 2s of TEMAV and water and with 5s of purge in between. At such growth conditions, ALD films are always amorphous as deposited and need annealing under correct conditions, with temperature and pressure having the most significant influence on polycrystalline film formation. Therefore, the ALD optimization strategy is mostly based on finding the correct pressure and temperature as well as time for the annealing step. While the temperature range for annealing appears similar to that used in the PLD process, the pressure range for the ALD process is much wider and was evaluated starting from 6.6 mTorr and finishing at 75 mTorr. Importantly, the time of annealing has played a very significant role in post ALD film crystallization. We have experimentally established that lower temperatures and longer times were absolutely critical in formation of ultrathin continuous ALD films (samples A1 and A2), while higher temperatures and shorter times were acceptable for thicker ALD films (samples A3–A5). Our ALD process optimization was initially based on our previous understanding of film formation during PLD growth. Indeed, it can be seen that Sample A1 in Figure 9 is resembling PLD films governed by dewetting behavior. To achieve a higher switching ratio, this ultrathin film was annealed in two steps, and the first step film with a lower thermal budget can be seen in Figure 8C. Similar to the evolution of the PLD sample (b), further annealing of the same film increased the thermal budget, decreased film surface energy, and correspondingly increased surface roughness also known as granulation of the film. Side by side comparison of this film after first and second annealing steps (Figure 8C,D) as well as more SEM demonstrating film behavior for different annealing conditions can be seen in Figure 8. Overall, the ALD film annealing process is in perfect agreement with the above-described physics of surface energy balance. Like in PLD growth, we have observed that film crystallization during annealing step is strongly dependent on the thickness of the deposited amorphous film. Depending on film thickness and kinetic energy delivered to the film through heating and pressure, nucleation points start to appear in the amorphous film (Figure 8B), if energy continues to increase, smooth films following dewetting behavior will form (Figure 8C,D) and if energy is yet increasing rougher granulated films will form (Figure 8E–H). Expectedly, a thinner film will require smaller energy for these film morphology transformations compared to thicker films. Evolution of the granulated film under yet increasing energy can be perfectly seen in Figure 8G where due to deposition discontinuity, an abrupt gradient in film thickness was formed. Even though this film received a fixed amount of kinetic energy for crystallization, we can see how the size of the grains is rapidly changing from below 100 nm to bigger grains that eventually start to disconnect and finally form big disconnected single grains of VO_2_ (from the top left corner to the bottom right corner of the image). All this variety of film morphologies are there due to the thickness gradient and is in absolute agreement with the law of surface energy balance and surface-energy minimization principle. Another clear example of this principle is a transformation of film (E) into film (F) after extra annealing time, Figure 8.

**Figure 8:**
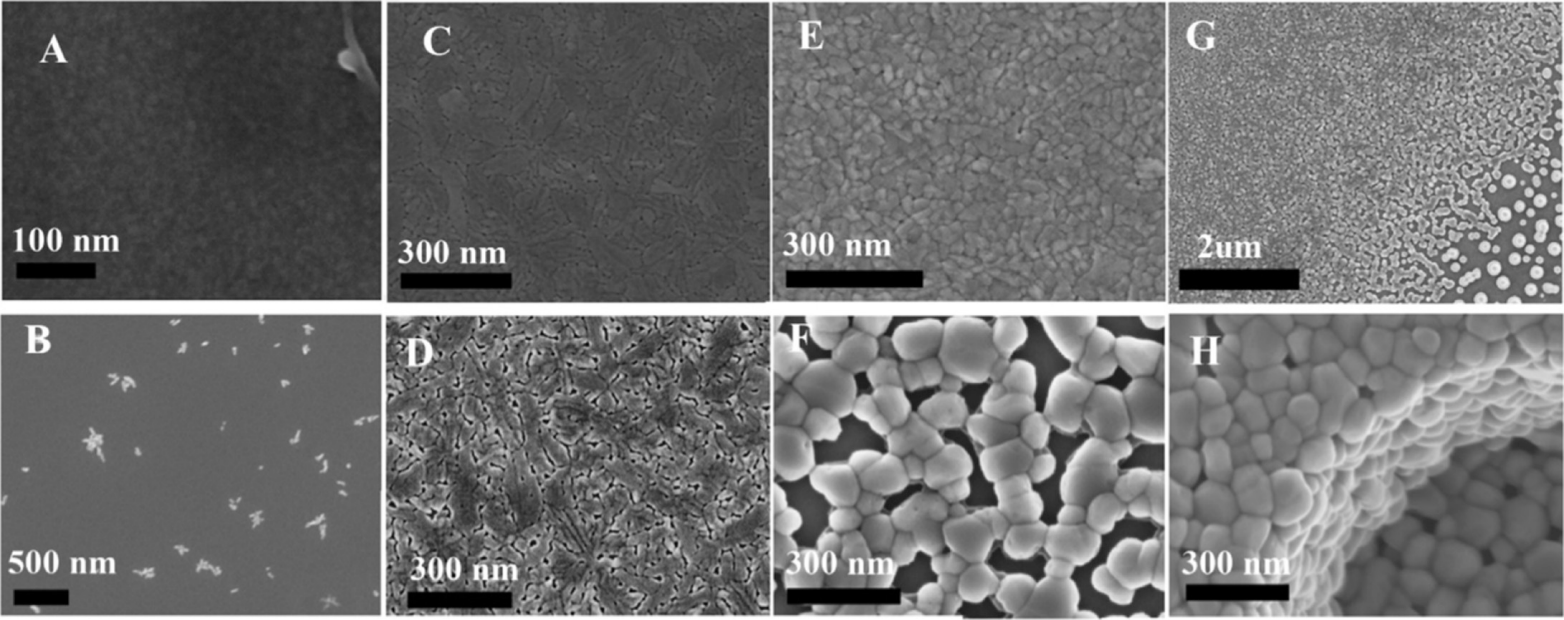
SEM images of VO_2_ films deposited by ALD under various annealing conditions. (A) As-deposited amorphous film; scale bar: 100 nm. (B) Film after 830 ALD pulses and annealing for 31 min at 430 °C under 75 mTorr; predominantly amorphous with a few nucleated flower-like islands; scale bar: 500 nm. (C) Film after 250 ALD pulses and annealing for 3 h 30 min at 400 °C under 6.6 mTorr; scale bar: 300 nm. (D) Same as (C) with an additional 30 min annealing at 450 °C under 6.6 mTorr; scale bar: 300 nm. (E) Film annealed for 25 min at 430 °C followed by 10 min at 450 °C under 18.7 mTorr; scale bar: 300 nm. (F) Same as (E) with an additional 20 min annealing at 450 °C under 37.5 mTorr; scale bar: 300 nm. (G) Film exhibiting a nonuniform thickness profile after annealing for 210 min at 450 °C under 6.6 mTorr; scale bar: 2 μm. (H) Film grown with 1500 ALD pulses and annealed for 75 min at 450 °C under 75 mTorr; 3D surface profile; scale bar: 300 nm.

To further investigate the optimization of the switching behavior in ALD-grown VO_2_ films, we selected five representative samples synthesized and annealed under varying conditions. Among these, the film processed at the highest annealing pressure of 75 mTorr exhibited the highest ON/OFF resistance ratio, highlighting the critical role of pressure in enhancing phase transition performance. Figure 9 shows all 5 successfully grown and annealed ALD samples, including the cross section of the best On/Off ratio sample, with corresponding thermal switching curves and XRD measurement showing the presence of a pure VO_2_ phase. Table 2 summarizes all input parameters for the ALD samples.

**Figure 9:**
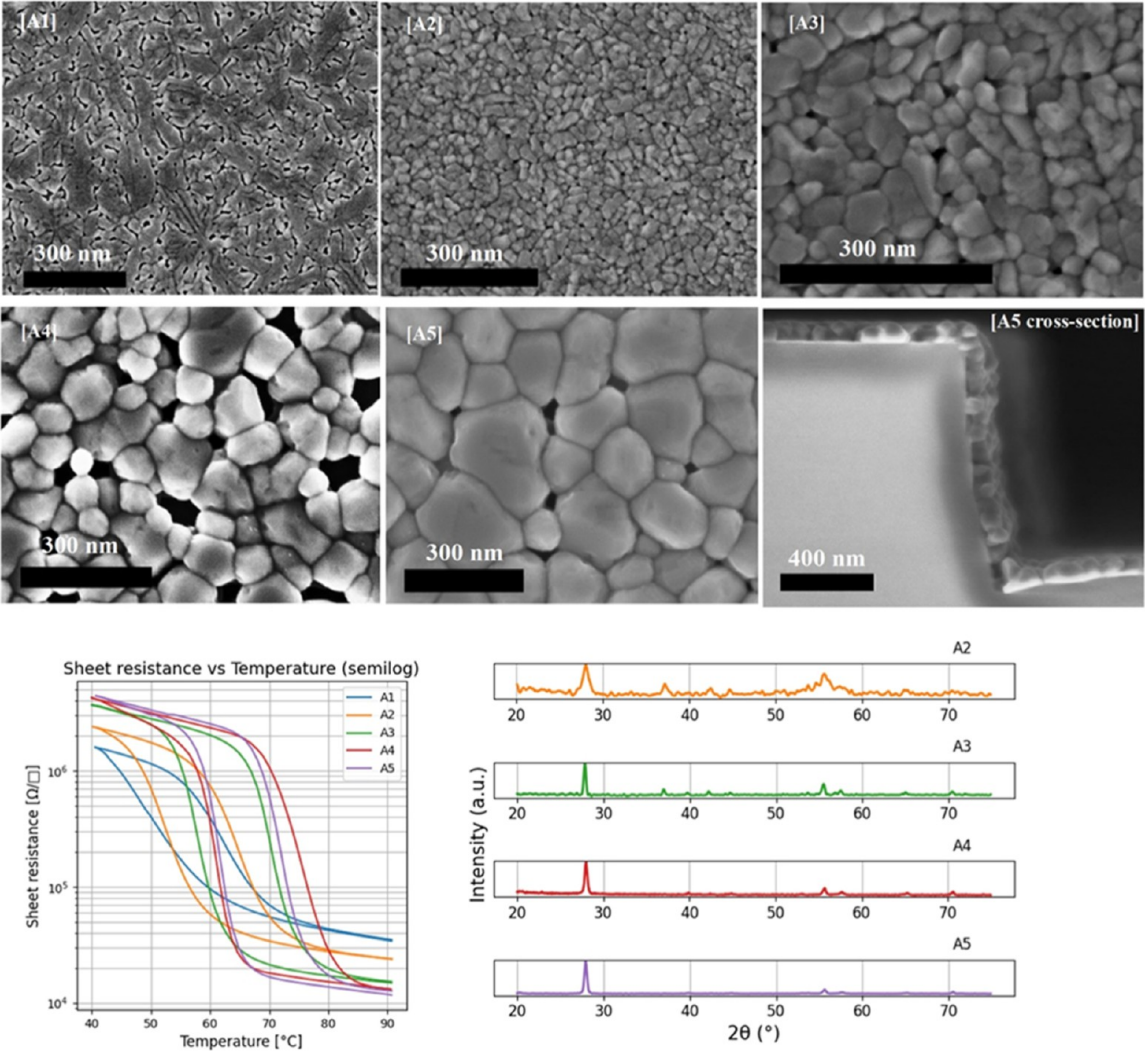
SEM micrographs A1-A5 and cross-section for A5 from left to right and respective thermal switching and XRD curves for A2-A5 ALD samples, sample A1 is not measured by XRD due to nonsufficient thickness of the film (only 6 nm). Scale bar SEM 300 nm for all except cross section 400 nm. Growth parameters for each sample can be found in Table 2.

**Table 2:** Summary of ALD-Grown VO_2_ Samples with Corresponding Growth Parameters, Film Thicknesses, ON/OFF Resistance Ratios, and Insulator-to-Metal Transition (IMT) Temperatures

sample	TEMAV [°C]	Chmb [°C]	# Puls	Ta [°C]	*T* [min]	*P* [mTorr]	*Z* [nm]	IMT [°C]	on/off
#A1	70	150	250	400/450	210/30	6.6	6	62.2	16.7
#A2	70	150	790	400/450	60/120	6.6	18	64.7	35
#A3	70	150	1500	470	90	6.6	30	70.3	78.6
#A4	70	150	1500	450	75	52	35	75.7	106
#A5	70	200	1500	450	75	75	40	72.2	136

Figure 10 demonstrates the experimentally observed dependency between VO_2_ film thickness and On/Off ratio as well as threshold IMT. These dependencies are very similar to those we have reported for the PLD-optimized recipe set of films with different thicknesses.

**Figure 10:**
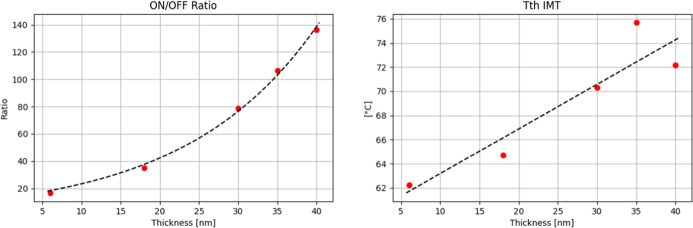
Thermal sweep results for ALD-grown VO_2_ samples A1–A5 (as listed in Table 2). Left: ON/OFF resistance ratio as a function of film thickness, showing an exponential fit. Right: Insulator-to-metal transition (IMT) threshold temperature as a function of film thickness, exhibiting a linear trend.

## Toward Ultrathin Films: Comparing PLD and ALD Films

One of the most interesting results achieved in this work is, indeed, our successful growth of an ultrathin switching polycrystalline VO_2_ film on a CMOS-compatible Si wafer with 200 nm wetox SiO2 with both PLD and ALD methods. In the case of PLD, we have achieved it by careful control of growth parameters, and scaling down the deposition time to the minimum allowed time on the instrument as described above. In the case of ALD ultrathin films, in addition to establishing that lower temperatures and longer times were absolutely critical in formation of ultrathin ≥30 nm films, while higher temperatures and shorter times were acceptable for ≥30 nm thicknesses, we were also inspired by the nonuniform-temperature deposition scheme suggested in Zhang et al. to increase nucleated island density. To further improve the switching behavior of ALD ultrathin films, we have adopted multistep annealing profiles, with the temperature, time, and pressure varying for each step (Samples A1 and A2 in Table 2). Figure 11 compares the microstructure of the ultrathin 8 nm PLD film grown with the optimized recipe and ultrathin 6 nm ALD film, as well as the optimized recipe PLD film of 40 nm and corresponding best ALD film of 40 nm. Figure 12 demonstrates a comparison of switching characteristics for the films from Figure 11. It can be noted that for both thicknesses, PLD has a superior On/Off ratio and smaller hysteresis. However, switching ratios for both ultrathin films are very close and big enough to be used in VO_2_-based devices.^33,34^ On the contrary, threshold IMT shows a significant shift due to the difference in grain sizes visible in Figure 8 and grain formation conditions. During PLD processes, the film is instantly polycrystalline and therefore grains are more strained from the beginning, while in the ALD process, films are completely amorphous at the deposition stage and turn into polycrystalline only during the annealing step, resulting in more relaxed films. The difference in the IMT can therefore be related to the differences in strain induced during crystallization of the film.

**Figure 11:**
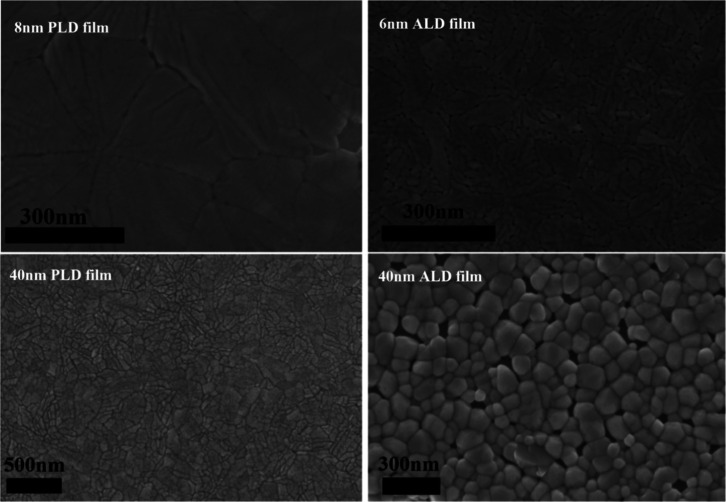
SEM images of the PLD and ALD VO_2_ thin films. Top: ultrathin 8 nm PLD and 6 nm ALD and Bottom: 40 nm thick PLD and ALD films.

**Figure 12:**
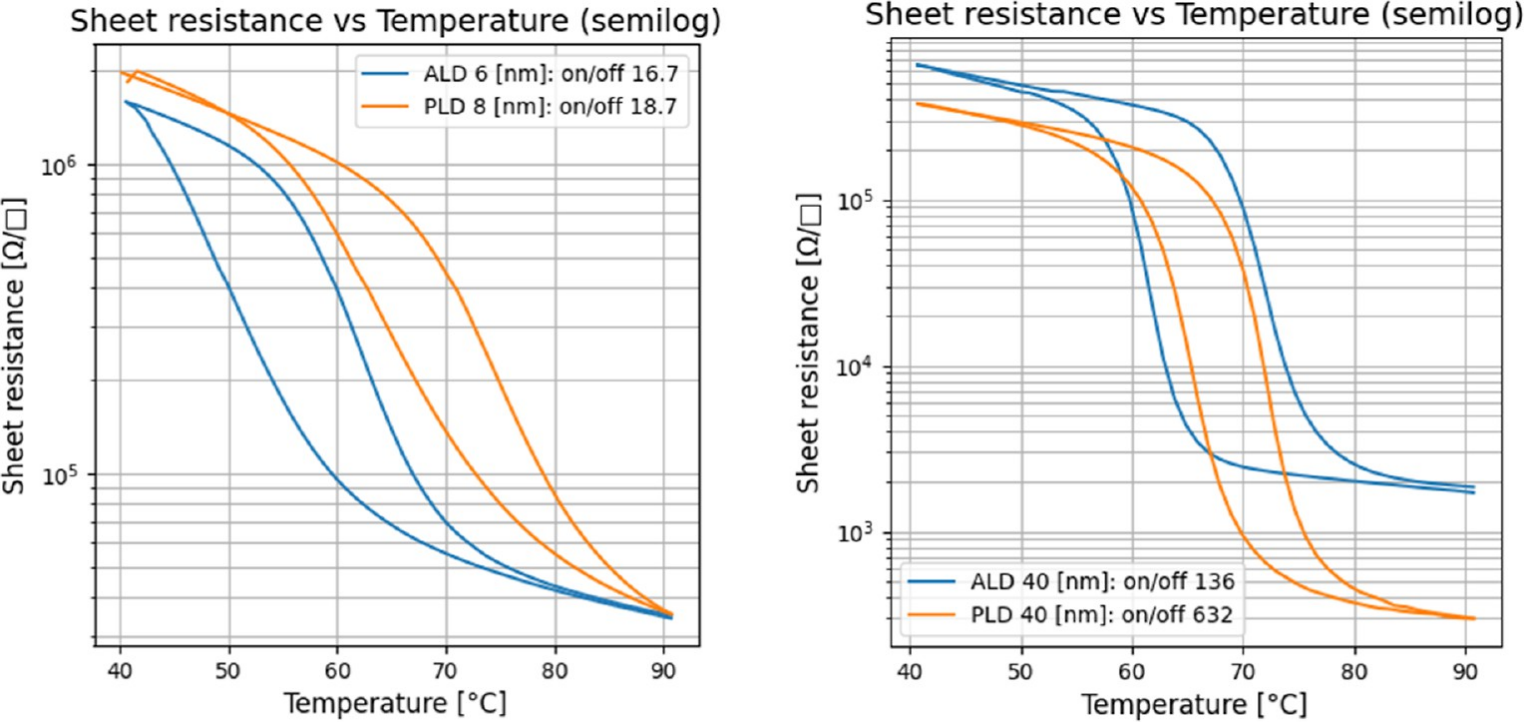
Comparison of thermal switching characteristics between ALD- and PLD-grown VO_2_ films. Left: Ultrathin films (6 nm ALD and 8 nm PLD). Right: Thicker films with comparable thicknesses (40 nm ALD and 40 nm PLD).

## Conclusions

In conclusion, this work offers a systematic, step-by-step analysis of the influence of growth parameters in PLD and ALD on the film formation and properties of CMOS-compatible polycrystalline VO_2_ films. These films, grown on standard Si/SiO_2_ wafers, were evaluated for electrical properties and surface morphology across a broad thickness range, from 6 to 200 nm. Through careful analyses of growth dynamics and optimization of growth conditions, continuous VO_2_ films with thicknesses below 10 nm were reproducibly grown on an industry-standard SiO_2_/Si substrate using both PLD and ALD methods.

The films were characterized using four-point probe measurements in a thermal chamber to assess the thermal switching behavior as well as SEM, XRD, and Raman spectroscopy for structural and morphological analyses. The insulator–metal transition (IMT) temperature exhibited thickness-dependent behavior, increasing with a reduced thickness in PLD-grown films and decreasing in ALD-grown films. Additionally, the On/Off switching ratio decreased with decreasing thickness in both methods.

Temperature and pressure during deposition and annealing were identified as the most critical factors influencing the On/Off ratio, MIT threshold, film morphology, and grain size distribution. The ability to achieve CMOS-compatible fabrication of VO_2_ films highlights the potential for seamless integration into existing silicon-based technologies, enabling the development of low-power, scalable devices for logic, memory, and neuromorphic applications. These findings provide a strong foundation for the design of vertically stacked devices in ferroelectric and memory applications.

## Abbreviations

PLD: pulsed laser deposition
ALD: atomic layer deposition
SEM: scanning electron microscopy
XRD: X-ray diffraction
MIT: metal–insulator transition
TEMAV: tetrakis ethylmethyl amino vanadium.
